# Fc-fusion proteins: new developments and future perspectives

**DOI:** 10.1002/emmm.201201379

**Published:** 2012-07-26

**Authors:** Daniel M Czajkowsky, Jun Hu, Zhifeng Shao, Richard J Pleass

**Affiliations:** 1 Key Laboratory of Systems Biomedicine (Ministry of Education) & State Key Laboratory of Oncogenes & Related Genes, Shanghai Jiao Tong UniversityShanghai, P. R. China; 2 Laboratory of Physical Biology, Shanghai Institute of Applied Physics, Chinese Academy of SciencesShanghai, P. R. China; 3 Liverpool School of Tropical MedicinePembroke Place, Liverpool, UK

**Keywords:** clinical tools, Fc-fusion proteins, Fc-receptors, immunoglobulins, therapeutic impact

## Abstract

Since the first description in 1989 of CD4-Fc-fusion antagonists that inhibit human immune deficiency virus entry into T cells, Fc-fusion proteins have been intensely investigated for their effectiveness to curb a range of pathologies, with several notable recent successes coming to market. These promising outcomes have stimulated the development of novel approaches to improve their efficacy and safety, while also broadening their clinical remit to other uses such as vaccines and intravenous immunoglobulin therapy. This increased attention has also led to non-clinical applications of Fc-fusions, such as affinity reagents in microarray devices. Here we discuss recent results and more generally applicable strategies to improve Fc-fusion proteins for each application, with particular attention to the newer, less charted areas.

## Introduction

**Figure 1:**
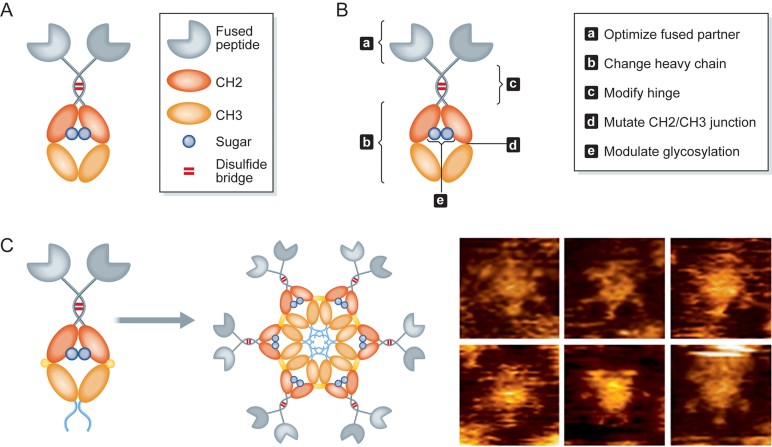
The structure of a prototypic IgG Fc-fusion and the means by which it can be presently modified Fc-fusions are homodimers in which an Fc domain of an antibody is covalently linked to another protein. The fusion partner is usually directly attached to the flexible hinge (as shown), the length and sequence of which varies between different IgG subclasses. Also shown is the approximate position of the N-linked oligosaccharides attached at Asn 297 in the IgG1 Fc-domain.There are a number of specific changes to the (monomeric) fusion construct that can be made to improve efficacy, as detailed in the text. A summary of these specific changes is included in Supporting Information Table S2.Fc-fusions can also be polymerized through engineered disulphide bridges localized to the CH2–CH3 junction and 18 amino-acid carboxy-terminal extensions known as tailpieces. The panel on the right shows atomic force microscopy images of hexameric hIgM-Fc fusions (attached to a malarial antigen) showing six-fold symmetric star-shaped complexes. The smaller radial projections are most likely the antigens (reproduced with permission; Mekhaiel et al, 2011b). Each panel is 75 × 75 nm^2^.

Fc-based fusion proteins are composed of an immunoglobin Fc domain that is directly linked to another peptide (Fig 1A). The fused partner can be any other proteinaceous molecule of interest, such as a ligand that activates upon interaction with a cell-surface receptor, a peptidic antigen (Ag) against a challenging pathogen or a ‘bait’ protein to identify binding partners assembled in a protein microarray. Most frequently though, the fused partners have significant therapeutic potential, and they are attached to an Fc-domain to endow the hybrids with a number of additional beneficial biological and pharmacological properties. Perhaps most important, the presence of the Fc domain markedly increases their plasma half-life, which prolongs therapeutic activity, owing to its interaction with the salvage neonatal Fc-receptor (FcRn; Roopenian & Akilesh, 2007), as well as to the slower renal clearance for larger sized molecules (Kontermann, 2011). The attached Fc domain also enables these molecules to interact with Fc-receptors (FcRs) found on immune cells, a feature that is particularly important for their use in oncological therapies and vaccines (Nimmerjahn & Ravetch, 2008). From a biophysical perspective, the Fc domain folds independently and can improve the solubility and stability of the partner molecule both *in vitro* and *in vivo*, while from a technological viewpoint, the Fc region allows for easy cost-effective purification by protein-G/A affinity chromatography during manufacture (Carter, 2011). In addition, most Fc-fusions are expressed as homodimers, and, as will be described later, can now be modified to polymerize into well-defined complexes containing twelve fused partners (Fig 1C). Thus an increase in avidity, and with it potency, from an isolated fused partner is also a significant advantage of Fc-fusion proteins.

Hence, as might be expected, the greatest focus of present work with Fc-fusions has been in therapeutic applications and there have been several recent notable successes (Table 1). Still, some of the most exciting advances with Fc-fusions will undoubtedly emerge from their more recent application in areas such as vaccines and microarray technologies. While much of this work has been focused on the specific details of the fused partner, there is also wide understanding of the significant tailoring that must be performed on the Fc-domain to maximize the effectiveness in each particular setting (Fig 1B; Strohl, 2009). In this perspective, we focus on recent results particularly related to the engineering of the Fc-domain for specific applications, and discuss potential future directions, with emphasis on the more novel, less investigated areas of application.

**Table 1:** Key Fc-fusion proteins and monoclonal antibodies (mAbs) in the clinic

Trade name (generic name)	Description	Indication of first FDA approval	Stage	Company
Fc-fusion				
Nulojix (belatacept)	CTLA-4 fused to the Fc of human IgG1	Organ rejection	FDA Approved (2011)	Bristol-Meyers Squibb
Eylea (aflibercept)	VEGFR1/VEGFR2 fused to the Fc of human IgG1	Age related macular degeneration	FDA Approved (2011)	Regeneron Pharmaceuticals
Arcalyst (rilonacept)	IL-1R fused to the Fc of human IgG1	Cryopyrin-associated periodic syndromes	FDA Approved (2008)	Regeneron Pharmaceuticals
NPlate (romiplostim)	Thrombopoietin-binding peptide fused to the Fc of human IgG1	Thrombocytopenia in chronic immune thrombocytopenic purpura patients	FDA Approved (2008)	Amgen/Pfizer
Orencia (abatacept)	Mutated CTLA-4 fused to the Fc of human IgG1	Rheumatoid arthritis	FDA Approved (2005)	Bristol-Meyers Squib
Amevive (alefacept)	LFA-3 fused to the Fc of human IgG1	Psoriasis and transplant rejection	FDA Approved (2003)	Astellas Pharma
Enbrel (etanercept)	TNFR fused to the Fc of human IgG1	Rheumatoid arthritis	FDA Approved (1998)	Amgen/Pfizer
mAbs				
Rituxan/MabThera (rituximab)	Chimeric mouse/human IgG1 targeting CD20	B cell lymphomas	FDA Approved (2006)	Biogen Idec/Genentech Hoffman-La Roche (Europe)
Herceptin (trastuzumab)	Chimeric mouse/human IgG1 targeting HER2	Breast cancer and gastroesophageal junction adenocarcinoma	FDA Approved (2006)	Genentech
Campath/Lemtrada (alemtuzumab)	Humanized IgG1 targeting CD52 on B and T lymphocytes	B cell chronic lymphocytic leukemia. In phase IIIa trials for multiple sclerosis	FDA Approved (2007)	Genzyme
Prolia/Xgeva (denosumab)	Fully human IgG2 targeting RANKL	Osteoporosis	FDA Approved (2010)	Amgen
Tysabri (natalizumab)	Humanized IgG4 tageting alpha-4 integrin	Multiple sclerosis and Crohn's disease	FDA Approved (2004)	Biogen Idec and Élan
Vectibix (panitumumab)	Fully human IgG2 targeting EGFR, ErbB-1 and HER1	Metastatic colorectal cancer (in patients with non-mutated KRAS	FDA Approved (2006)	Amgen
Soliris (eculizumab)	Humanized IgG2/4κ targeting complement protein C5	Paroxysmal nocturnal haemoglobinuria to reduce haemolysis	FDA Approved (2007)	Alexion Pharmaceuticals
Erbitux (cetuximab)	Chimeric mouse human IgG1 targeting EGFR, ErbB-1 and HER1	Metastatic colorectal cancer (in patients with non-mutated KRAS	FDA Approved (2006)	Bristol-Myers Squibb and Eli Lilly
Avastin (bevacizumab)	Humanized IgG1 targeting VEGF	Metastatic colorectal cancer and HER2-negative metastatic breast cancer	FDA Approved (2008) Withdrawn (2011)	Genentech/Roche
Remicade (infliximab)	Chimeric mouse human IgG1 targeting TNF-α	Psoriasis, Crohn's disease, ankylosing spondylitis, rheumatoid arthritis	FDA Approved (1998)	Janssen Biotech/Schering-Plough

## Fc-fusion proteins as drugs

As of the end of 2011, six Fc-fusion based drugs were on the market, with four in phase 3 clinical trials and many others at different stages of pre-clinical development (Table 1). The advantages of Fc-fusion drugs over other types of biopharmaceuticals have been extensively reviewed recently (Beck & Reichert, 2011; Nelson & Reichert, 2009; Strohl, 2009; Strohl & Knight, 2009). In terms of market value, Fc-fusion proteins and monoclonal antibodies (mAbs) together account for 43% of all therapeutic proteins based on published sales in 2008 (Strohl & Knight, 2009), and examination of those in the clinical pipeline suggests that this market share is likely to rise still further (http://clinicaltrials.gov/). In fact, global sales of the most successful Fc-fusion, etanercept, with USD $7.3 billion in 2010, exceeded the most successful therapeutic IgG (bevacizumab) with USD $6.9 billion (Beck & Reichert, 2011). With such commercial successes, Fc-fusions now represent 20% of all antibody-based medicines with FDA approval, fuelling the development of second- and third-generation versions, examples of which are discussed below.

Most of these Fc-fusions target receptor-ligand interactions, working either as antagonists to block receptor binding (*e.g.* etanercept, aflibercept, rilonacept, belatacept, abatacept) or as agonists to directly stimulate receptor function to reduce (*e.g.* alefacept) or increase immune activity (*e.g.* romiplostim). These are also common targets for therapeutic mAbs (Reichert, 2011), which have been studied for a much longer period of time than Fc-fusions, although they can actually be considered as a particular type of Fc-fusion construct (Table 1). Evidence from studies with therapeutic mAbs may therefore usefully inform on how improvements to Fc-fusion proteins can be made. As will be made clear, the desired effect of these medications and the range of interactions with Fc effector systems are intimately linked.

### Increasing effector function

Many therapeutic mAbs (*e.g.* rituximab, trastuzumab, alemtuzumab) function by targeting cancer cells for destruction by natural killer (NK) cells through antibody-dependent cell-mediated cytotoxicity (ADCC; Table 1), a cytolytic effector mechanism believed owing to Ag-specific IgG1 binding FcγRIIIA localized on the NK cells (Congy-Jolivet et al, 2007; Strohl, 2009). The absolute requirement for NK cells is however coming under scrutiny as *in vivo* work in mouse models also implicates monocytes/macrophages as important effector cells (Biburger et al, 2011). Still, patients with high affinity FcγRIIIA variants respond better to therapy (Veeramani et al, 2011) and interactions with this receptor are thought critical for ADCC (Strohl, 2009). Enhancing the affinity of mAbs for FcγRIIIA was therefore expected to improve tumour killing through ADCC. This was subsequently achieved by modifying the amino acid sequence in the Fc domain or by de-fucosylation of the N-linked oligosaccharides on the Fc region (Shinkawa et al, 2003; Stavenhagen et al, 2008). Such modifications have also been shown to improve the therapeutic potential of clinically relevant Fc-fusion proteins, probably for similar reasons (Shoji-Hosaka et al, 2006). It should be noted though that some mAbs and Fc-fusions function by additional mechanisms than ADCC, such as apoptosis (Peipp et al, 2008), and whether such modifications also improve the efficacy with these drugs remains to be investigated.

**Glossary**

**ADCC (antibody-dependent cell-mediated cytotoxicity)**

A cytotoxic reaction in which FcR-bearing killer cells recognize target cells via specific antibodies.

**Avidity**

The association constant for multivalent binding by the Fc, distinguished from affinity, which is determined by the binding strength of a single Fc interaction.

**CDC (complement-mediated cytotoxicity)**

The interaction of complement proteins found in blood with opsonized antibodies (IgG and IgM) leading to the activation of the classical pathway and resulting in the killing of pathogens or tumour cells by lysis.

**Dendritic cell**

A professional immune cell so named after their dendritic morphology. Capable of delivering Ag and potent stimuli to T cells during immunization with vaccines.

**Fab**

Fragment with Ag binding specificity. Part of the Ab molecule consisting of the light chain and the NH_2_-terminal half of the heavy chain held together by an inter-chain disulphide bond.

**Fc**

Fragment crystallizable. Part of the Ab molecule that interacts with FcRs. Consisting of the carboxy-terminal heavy chains disulphide bonded to each other through the hinge region.

**Fc-receptors**

Cell surface and intracellular molecules that bind the Fc region of Ab. For IgG, these FcγRs can be both activating, *e.g.* FcγRI, or inhibitory, *e.g.* FcγRIIb. Some FcRs, *e.g.* Fcα/µR can bind more than one class of Ab. Biological activation results from cross-linking and aggregation of immunoreceptor tyrosine-based activation (ITAM) or inhibitory (ITIM) motifs in their cytoplasmic sequences.

**Fc-receptor-like (FcRL) proteins**

A family of cellular receptors homologous to FcγRI and predominantly expressed by B cells. They function to co-stimulate, or inhibit, B cell receptor signalling through concensus ITAMs and ITIMs. Unlike the classical FcRs, FcRL4 (for IgA) and FcRL5 (for IgG) are two members of the FcRL family that bind monomeric immunoglobulin poorly, and are likely to be important for immune-complex dependent human B cell regulation. They may therefore constitute target receptors on B cells for immune-complex mediated vaccination.

**Immune-complexes**

Protein complexes formed by the binding of antibodies to soluble Ags. They can be both activating and/or inhibitory, a property most likely influenced by their overall size and the class of antibody found within the complex.

**Intravenous immunoglobulin (IVIG)**

A highly pure preparation of immunoglobulin prepared from healthy donors. IVIG is licensed for the treatment of ITP, Guillain–Barré syndrome, chronic inflammatory demyelinating polyneuropathy and Kawasaki disease, but is increasingly being used in the treatment of other autoimmune diseases.

**Natural killer cell (NK)**

A type of large granular and cytotoxic immune cell involved in killing intracellular pathogens (particularly viruses) and tumours. They do not possess variable receptors for Ag but have many other receptors through which they can recognize and kill tumours or virus-infected cells.

**Neonatal FcR (FcRn)**

A receptor for the transport and maintenance of IgG in the circulation having a structure resembling MHC Class I molecules.

**Sialic acid**

A sugar that often occupies the terminal position within the oligosaccharide attached to IgG at Asn297 (Fig 1). It is bound by inhibitory receptors, *e.g.* sialic acid binding Ig-like lectins (Siglecs) that are implicated in the mode of action of IVIG.

Another common mode of action of anti-tumour mAbs is complement-dependent cytotoxicity (CDC), in which direct interaction of surface Ag-bound Abs with complement C1q triggers cell death through the complement cascade (Idusogie et al, 2001; Weiner, 2010). The finding that complement regulatory proteins secreted by tumours can significantly decrease the CDC activity of rituximab (Treon et al, 2001), in fact necessitated the search for Ab therapeutics with improved CDC activity (Idusogie et al, 2001), although it should be noted that the mechanism of action of rituximab appears complex (Weiner, 2010). Most Fc-fusions studied to date do not bind C1q and are unable to activate the ensuing complement cascade. This inability of Fc-fusions to activate complement probably stems from the lack of Fab residues in the fusion that contribute to interactions of intact IgG with C1q (Gaboriaud et al, 2003). The development of Fc-fusions with potent CDC activity thus would probably require the identification of mutations solely within the Fc region that could compensate the lack of Fab-domain contact.

### Decreasing effector function

For some therapeutic applications however, activation of complement and FcγRs are best avoided. For example, it is not desirable to stimulate complement during the neutralization of pathogenic molecules, such as cytokines, and Fc-fusions have been found useful to this end (Capon et al, 1989; Hoffman et al, 2008; Peppel et al, 1991). At the moment, all Fc-fusions licensed for clinical use have the IgG1-Fc domain, which can bind with moderate to high affinity to FcγRs (Bruhns et al, 2009). Owing to the relative lower affinity of human IgG2 and IgG4 towards FcγRs and complement receptors (Bruhns et al, 2009), these two subclasses are being developed as therapeutic mAbs (*e.g.* denosumab, natalizumab, panitumumab and eculizumab) for those cases where minimal effector potential is required (Table 1). The use of the IgG2 and IgG4 Fc domain in fusion proteins has yet to reach the clinic, although a number of studies have suggested that they may have superior properties (Cox et al, 2004; Kumar et al, 2007; Soltani et al, 2007). In one case, glucagon like peptide 1 (GLP-1) was fused to human IgG2 to avoid unwanted immunogenicity and was shown to have superior therapeutic and pharmacologic properties to native GLP-1 in a mouse model of type I diabetes (Wang et al, 2010).

However, it is also possible to produce a decreased effector function of one effector system by the simulataneous activation of another effector system. In particular, the critical observation that FcεR activation of mast cells can be downregulated by the simultaneous activation of inhibitory FcγRIIB (Daeron et al, 1995), has stimulated the development of a number of Fc-fusion proteins that inhibit inflammation, and which may have particular application in treating allergic asthma (Supporting Information Table S1). One such construct, a double fusion of the Fc domain of IgG1 to the Fc domain of IgE is currently under development at Biogen-Idec for use as a long-term systemic therapy for the treatment of IgE-mediated disease, including severe food allergy (Saxon et al, 2008; Van Scott et al, 2008). In the case of Fc-fusions to the IgE-Fc domain, how injected monomers compete for binding with *in vivo* IgE already bound to the high affinity FcεRI (or FcγRI in the case of IgG) remains to be fully investigated, although strategies that increase their valency (see below), thereby increasing their overall functional affinity, may prove useful if this competition is significant.

### Improving pharmacokinetics of Fc-fusions

One way to improve the therapeutic efficacy of Fc-fusions more generally is to increase their plasma half-life, which is primarily mediated through interactions with FcRn (Roopenian & Akilesh, 2007). The site of interaction of IgG with FcRn has been localized to the CH2–CH3 domain interface (Martin & Bjorkman, 1999), and mutations have been identified in this region that increase the affinity of IgG for FcRn at acidic but not neutral pH (Dall'Acqua et al, 2006; Yeung et al, 2009). Incorporation of such mutations into two therapeutic mAbs (cetuximab and bevacizumab) not only increased their *in vivo* half-life but significantly improved tumour killing as a consequence (Zalevsky et al, 2010). The half-life of Fc-fusions is typically shorter than intact Abs (1–2 weeks against 3–4 weeks; Suzuki et al, 2010), and so it might be expected that equivalent mutations would make a more significant effect on the half-lives and overall efficacy of Fc-fusions than mAbs. Interestingly, the observation of pulmonary delivery of an erythropoietin Fc-fusion protein in non-human primates via FcRn indicates that this receptor can also be harnessed to non-invasively deliver bioactive proteins into the systemic circulation in therapeutic quantities, a finding that also impacts on their use as vaccines (see below; Bitonti et al, 2004).

### Optimizing the fused partner can be critical

While modifications to the Fc-domain such as those described above can generally improve therapeutic function, it should be noted that subtle binding properties of the fused partner also make a dominating contribution to the ultimate activity of the biologic in the clinical setting. For example, both etanercept (Fc-fusion) and infliximab (intact mAb) neutralize soluble tumour necrosis factor (TNF)-α, but only infliximab is capable of inducing a clinical response in Crohn's patients, although both drugs significantly improve rheumatoid arthritis (Sandborn et al, 2001). Direct comparisons of the two drugs have shown that only infliximab is able to bind activated lymphocytes, induce apoptosis, and activate caspase 3 (Van den Brande et al, 2003). These differences may be owing to differences in the affinity (Van den Brande et al, 2003) or stoichiometry (Horiuchi et al, 2010; Scallon et al, 2002) of these drugs for transmembrane TNF-α, which is expressed by activated lymphocytes (Van den Brande et al, 2003). Hence, identification of the optimal binding properties of the fused partner for its intended receptor may prove an essential step in the development of the drug for a desired clinical activity.

### Novel Fc-fusion platforms for therapeutic applications

In addition, whether or not the fused partner even retains its biological activity when attached to an Fc domain depends on many factors that need to be determined for each molecule. In order to improve the chances for a stable fused partner, for some proteins, it may be necessary to include a ‘chaperone’ protein whose presence stabilizes the desired partner, as recently demonstrated for a Toll-like receptor 4 Fc-fusion (Jung et al, 2009).

**Figure 2:**
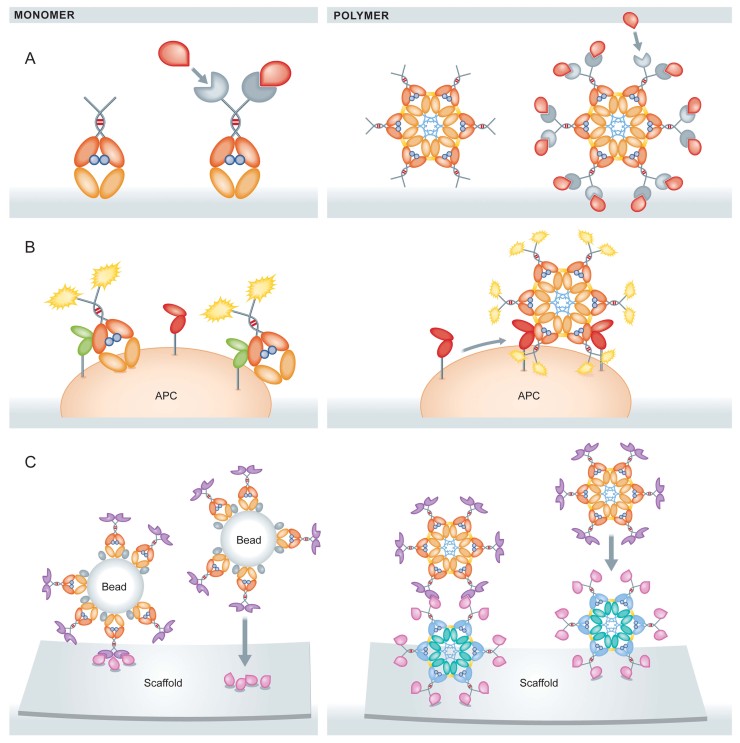
Different capabilities for different Fc-fusion stoichiometries Polymeric Fc-fusions are expected to greatly increase the effectiveness of therapeutic fusions that function either by binding free ligands (such as etanercept) or by interacting with carbohydrate-receptors in IVIG (for complexes without the fusion partner), as a consequence of their greater valency. Monomeric versions would be expected to exhibit more rapid tissue penetration than the polymers, although both can likely access similar regions (Kaveri et al, 2012; Vollmers & Brandlein, 2006).Monomeric and polymeric Fc-fusions interact with fundamentally different sets of FcRs. The clustering of low-affinity FcRs on APCs by polymeric Fc-fusions would initiate signals required by APCs for their maturation and development, essential steps in effective vaccination.Monomeric Fc-fusions can be coupled to protein G/A coated micron-sized beads to high, though variable, stoichiometry for the detection of critical low-affinity protein:protein or protein:drug interactions in protein microarrays. By comparison, polymeric Fc-fusions as nanosized Fc-scaffolds might be more suitable to sensitive *in vivo* assays where heavy beads are inappropriate.

Another concern that is undoubtedly common to many constructs is whether the target molecule can bind with sufficient affinity to its cognate protein when situated in the homodimeric Fc-fusion architecture (see Fig 1A). One way this may be overcome is by engineering multiple specificities and/or avidity into the Fc-fusion construct (Figs 1C and 2A). For the former, partners that can bind by more than one site are less likely to be perturbed at all of the sites in the fusion construct, and for the latter, an increase in the number of partners would enable significant binding even for more transient, lower-affinity interactions. The development of heterodimeric Fc platforms based on strand-exchange engineered domains (SEED) CH3 heterodimers composed of alternating segments of human IgA and IgG CH3 sequences may allow for the development of multiple specificities within the existing homodimeric Fc-fusion platform (Muda et al, 2011).

Multiple avidity has very recently been engineered into Fc-fusions to generate both tandem Fc repeated homodimers (Nagashima et al, 2011), and star-shaped hexameric constructs (Mekhaiel et al, 2011b), that suggest it may be possible to generate non-immunogenic drugs that can bind up to twelve target molecules (Fig 2A). However, one of the most prominent arguments against the use of polymeric Abs, particularly in oncology applications, is that their larger size prevents penetration into tissues. Although polymers naturally show a slower penetration time, even intact IgM can reach implanted tumours and metastases in patients after *i.v.* or *i.p.* administration (Kaveri et al, 2012; Vollmers & Brandlein, 2006), and IgM now represents an attractive vehicle for the delivery of IgM-conjugated drugs into leukemic B cells expressing high levels of FcµR (Vire et al, 2011). Slower penetration and accumulation may even be an advantage for the transduction of stronger pro-apoptotic or survival signals to cell surface receptors (Peipp et al, 2008), since natural IgM plays a pivotal role in the clearance of apoptotic and altered cells through CDC, inhibition of inflammation, removal of misfolded proteins, and in the regulation of auto-reactive B cells (Kaveri et al, 2012). Polymer–drug conjugates are also increasingly being explored as nanosized medicines (Canal et al, 2011) and approaches that increase multivalency may therefore have particular relevance to the growing number of therapeutic antibody fragments entering the clinic, such as the dromedary V_HH_ minibodies (Nelson, 2010), single-chain Fv based anti-human immune deficiency virus (HIV) proteins (West et al, 2012) or non-antibody based protein scaffolds (Lofblom et al, 2011).

We also note that recent studies indicate that IgA (Bakema et al, 2011; Bakema & van Egmond, 2011b; Lohse et al, 2011), IgE (Karagiannis et al, 2011) and IgM (Ammann et al, 2012; Kaveri et al, 2012) may also serve as alternatives to the classic IgG backbone for therapeutic Fc-fusions. While these studies utilized these different backbones to target specific effector systems, there may also be beneficial effects to the activity of the fused partner within the different architectures that should also be explored.

## Fc-fusion proteins as vaccines

### Choice of Fc-scaffold is critical

The possibility to directly target FcRs with the fused partner holds the promise for Fc-fusion proteins as vaccines (Supporting Information Table S1). While for therapeutic applications, the focus is on enhancing or inhibiting interactions with a narrow subset of FcRs, for vaccination applications, the focus is more on identifying which FcRs are to be targeted on which Ag presenting cells (APCs). Since most APCs only express FcγRI, FcγRIIA, FcγRIIB, FcεRI, FcεRII, FcRL4 and FcRL5 on their surface (Bajtay et al, 2006; Wilson et al, 2012), it is expected that the Fc domains that bind optimally to one or more of these receptors will make better Ag delivery vehicles. For example, most dendritic cell (DC) subsets, including Langerhans cells in the skin (a common site for vaccine delivery), do not express FcγRIII (CD16) or FcγRI (CD64), and therefore Fc-fusions optimized for delivery of Ag to FcγRIIA/FcγRIIB may be more favourable for vaccine approaches. The counterintuitive observation that inhibitory FcγRIIB, rather than activating FcγRs, are required for enhancing adjuvant and anti-tumour activity of agonistic CD40 IgG1 mAbs supports this assertion (Li & Ravetch, 2011). Furthermore, the finding that some activating FcγRs may also deliver inhibitory signals has made identifying individual FcγRs involved in effective immunization difficult (Pfirsch-Maisonnas et al, 2011). The optimal use of Fc-fusions in vaccines will therefore be predicated on delivering the fine balance required between ‘activatory’ and ‘inhibitory’ functions of FcRs.

These waters have recently been muddied by the discovery of two members of the FcRL family FcRL4 and FcRL5, potential inhibitory receptors expressed on the surface of mature human B cells that uniquely bind human IgA and IgG immune-complexes, respectively (Wilson et al, 2012). The ability of these receptors to bind complexed antibody, along with their known ability to regulate B cell Ag receptor signalling, suggests an important role for these newly identified receptors in Fc-fusion development, either as drugs or vaccines (Wilson et al, 2012). It should also be noted that the choice of FcR to target in vaccines will not solely be determined by surface availability of the FcR on APCs, as an increasing number of intracellular Fc-binding proteins are being discovered that also influence the development of protective immunity, including FcRn (Roopenian & Akilesh, 2007), FcRLA (Santiago et al, 2011; Wilson et al, 2010) and TRIM21 (McEwan et al, 2011).

Human IgG3 activates complement and FcγR-mediated functions more effectively than any other IgG subclass (Bruhns et al, 2009; Salfeld, 2007), yet most Fc-fusions presently studied in vaccines include only the human IgG1 backbone (Table 1 and Supporting Information Table S1). Human IgG1 and IgG3 bind equally well to FcγRIIB, although a recent analysis of specificity and affinity interactions implies that human IgG1 is better at engaging FcγRIIA than human IgG3, which binds more efficiently to FcγRIIIA (Bruhns et al, 2009). This suggests that human IgG1 based Fc-fusions would indeed be the preferred backbone to deliver Ag to APCs. Use of the IgG3-Fc domain in fusions would also have to overcome technical difficulties, such as its susceptibility to proteolysis (Jefferis, 2007) and documented shorter half-life (Morell et al, 1970). The latter results from the presence of an arginine at position 435 in the most commonly studied allotype of human IgG3 that significantly reduces the rescue efficiency by FcRn (Stapleton et al, 2011). By contrast, the histidine 435 containing IgG3 allotype has a half-life in humans comparable to IgG1 and also gives enhanced protection against a pneumococcal challenge in mice (Stapleton et al, 2011). If the other technical challenges could also be overcome, the IgG3 Fc-domain, with its longer hinge region, might serve as a necessary alternative in those cases where the fused Ag is found to block binding of the IgG1-based fusion to target FcRs (as described below; Mekhaiel et al, 2011b).

However, in one recent example where this was not a problem, IgG1-Fc-fusions were shown to induce potent and protective immune responses to virulent herpes simplex virus (HSV; Ye et al, 2011) and HIV (Lu et al, 2011), when administered intranasally in the presence of CpG, an adjuvant used to overcome possible mucosal tolerance. This immunization strategy was critically dependent on FcRn and induced both mucosal and systemic Ab, but more importantly, triggered potent and life long memory responses. These results suggest that mutations that enhance binding to FcRn, as described above, may also increase the potentcy of human IgG1 based Fc-fusions in vaccines.

Such mutations however are also likely to influence antibody-dependent intracellular neutralization via TRIM21, since both FcRn and TRIM21 bind IgG at the CH2–CH3 domain interface (McEwan et al, 2011; Roopenian & Akilesh, 2007; Fig 1A). TRIM21 has been shown to direct IgG-opsonized viruses to the ubiquitin–proteasome system for disposal (McEwan et al, 2011) but is also involved in processing Ag for cross-presentation by major histocompatibility complex (MHC) class I (McEwan et al, 2011). The recent finding that mouse CD8^−^CD11b^+^ but not CD8^+^CD11b^−^ DCs also require FcRn to cross-present IgG-containing immune-complexes (ICs) suggests that FcRn and/or TRIM21 targeting strategies may also need to be carefully considered for vaccines (Baker et al, 2011). Whether or not different human DC subsets also differentially express FcRn and TRIM21 remains to be determined (Zhu et al, 2001), although transgenic mice overexpressing bovine FcRn have been shown to develop more potent humoral responses to weakly immunogenic Ags (Cervenak et al, 2011).

### Alternative Fc-scaffolds should be explored

Some FcRs found on adaptive immune cells may make better targets for Fc-fusions based on antibody classes other than IgG. For example, in the aforementioned monomeric Fc-fusion HSV and HIV studies (Lu et al, 2011; Ye et al, 2011), the IgG-Fc may not be appropriate for generating mucosal secretory IgA responses for the control of mucosal pathogens. It may be more appropriate to fuse Ags to the Fc domain of IgA, since IgA-immune complexes (ICs) can induce potent proinflammatory responses that lead to protection from mucosal pathogens, including *Mycobacterium tuberculosis* (Bakema & van Egmond, 2011a; Balu et al, 2011). Monomeric interactions of IgA with FcαR can trigger equally potent inhibitory and apoptotic signals that block activation via other receptors suggesting that monomeric IgA Fc-fusions may be better developed for anti-inflammatory applications at mucosal sites (Bakema & van Egmond, 2011a). The recent finding that high plasma concentrations of IgA to HIV-1 Env inhibit protective responses mediated by IgG is supportive of an inhibitory role played by plasma IgA (Haynes et al, 2012), a phenomenon that has been observed with other pathogens (Griffiss & Goroff, 1983; Shi et al, 2011), in the regulation of autoantibodies (Quan et al, 1998), and in cancer (Mathew et al, 1981) or idiopathic thrombocytopenic purpura (ITP) (Arnason et al, 2012). The recent finding that heat-aggregated IgA can bind to inhibitory FcRL4 abundantly expressed by mucosal memory B cells suggests a possible mechanism (Wilson et al, 2012). It may thus be possible to design Fc-fusions based on IgA to limit B cell activation against chronic pathogens, self-Ag or commensal flora. Since down-regulation of FcRL4 in ‘exhausted’ memory B cells from HIV patients enhances their responsiveness to HIV (Kardava et al, 2011), reagents that block FcRL4 may lead to renewed generation of neutralizing Abs for HIV and other chronic viral infections, particularly if they can be complexed into polymers.

IgM may also be an alternative candidate for fusion technology since it forms natural polymers that may mimic the ‘depot-effect’ of adjuvants (Czajkowsky et al, 2010; Leroux-Roels, 2010), binds C1q and TRIM21 (McEwan et al, 2011), is involved in Ab subclass switching (Kaveri et al, 2012; Rapaka et al, 2010), shapes the ensuing immune response (Rapaka et al, 2010), and has been shown to be an excellent natural adjuvant in vaccines (Harte et al, 1983; Stager et al, 2003). IgM has the added advantage of engaging unique receptors on B, T (CD4^+^/CD8^+^) and NK lymphocytes that may stimulate memory responses required by efficacious vaccines (Kaveri et al, 2012; Vire et al, 2011).

IgE receptors are also expressed by APCs including most DC subsets (Bajtay et al, 2006), suggesting that Fcε-fusions may be usefully explored in vaccine approaches. Although Fcε-fusions to Ags have yet to be made, studies with IgE mAbs (Reali et al, 2001), or mini-membrane Fcε (Nigro et al, 2012), suggest that they may be particularly useful in cell-based tumour vaccines. Whether such approaches can be safely translated to the clinic without the risk of anaphylaxis must be fully investigated before IgE Fc-fusions can be used in general (Van Scott et al, 2008).

### Valency of Fc-fusion is critical

Monomeric Fc-fusion proteins would not be expected to cross-link multiple low-affinity receptors on APCs, which are required for the enhanced cell signalling necessary for a well-balanced immune response (Fig 2B). With the exception of FcγRI, most FcγRs, including FcRL5, FcγRIIA and FcγRIIB, that are needed to generate protective immune responses by APCs (Bruhns et al, 2009; Li & Ravetch, 2011; Nimmerjahn & Ravetch, 2008), can only interact with high avidity to Abs or Fc-fusions presented to the immune system as ICs (Getahun & Heyman, 2006; Wilson et al, 2012). ICs are potent activators of DCs initiating vigorous immune-responses even in the presence of inhibitory FcγRIIB (Getahun et al, 2004) and can cross-prime MHC Class I and II leading to optimal CD4^+^ and CD8^+^ T-cell responses (Regnault et al, 1999).

Making well-defined preparations of native ICs with which to study their therapeutic and vaccination potential is impractical. The optimal ratio of Ab:Ag to use in generating ICs, and the fact that they are highly unstable, generates significant obstacles for any rational investigation. A number of approaches have been explored to engineer monomeric Fc-fusions to improve avidity binding to APCs (Jensen et al, 2007; Nagashima et al, 2011). In one case, human serum albumin (HSA) was fused to multiple linear Fc repeats of human IgG1 (Jensen et al, 2007). Although, only capable of delivering two molecules of HSA, this monomeric construct nonetheless elicited significant B and T cell proliferative responses in mice in the presence of adjuvant, suggesting that linear monomeric Fc-fusions that cross-link multiple FcγRs can induce potent immune responses. Although this finding is confounded by the ability of HSA to interact directly with mouse FcRn (Roopenian & Akilesh, 2007; Stapleton et al, 2011), and by a requirement for adjuvant, it has been extended to modified botulinum neurotoxin fusions (Supporting Information Table S1). Since linear multiple Fc constructs bound C1q weakly (Jensen et al, 2007), the reason for their improved immunogenicity must derive from a combination of FcγR cross-linking together with non-specific effects mediated by co-administration of adjuvants.

An alternative approach to generate polymeric human IgG Fc-fusion proteins has recently been described, involving the use of multimerizing protein tags (Fig 1C; Mekhaiel et al, 2011b). With these contructs, well-defined hexameric Fc-fusion complexes containing twelve fused partners were produced to high yield and purity. Analysis of their affinity to FcγRs and FcRn showed a significantly increased binding strength over monomeric versions, although only in those constructs without the particular fusion Ags. With the fused Ags present, the complexes exhibited a reduced ability to interact with FcγRs, FcRn and complement, which was also reflected in their significantly lower immunogenicity than the monomeric or dimeric counterparts (Mekhaiel et al, 2011b). The use of IgG3 which has an extended hinge-region, or the use of other molecular extensions (SEEDbodies; Davis et al, 2010) or alternative multimerizing tags (Rossi et al, 2009; Wright, 2009), may allow for the design of polymeric Fc-fusions that do interact with high avidity to FcγRs without the perturbative effects from the fused Ag. Alternatively the use of smaller Ags, peptides, mimotopes or mimetibodies incorporating B and T cell epitopes may prove more effective for use in vaccine approaches where multivalency is a requirement (Gil et al, 2009; Huang, 2009).

### Choice of antigen is critical

In the aforementioned HSV and HIV studies, how monomeric Fc-fusions cross-link surface FcγRs on mucosal APC was not investigated, although HSV-gD (the fused peptide used in Ye et al, 2011) is known to bind directly to TNFRSF14 expressed on the surface of B and T lymphocytes (Heldwein & Krummenacher, 2008) and Gag proteins (the fused peptide in Lu et al, 2011) are targeted to the plasma membrane of most haematopoietic cell types (Saad et al, 2006). This suggests that cross-linking could be achieved by monomeric Fc-fusions if these receptors are found on the same APCs that express FcγRs. Such a strategy may not be appropriate for all Ags, especially those that do not interact directly with APCs or can interfere with the Fc (Wines et al, 2012). Indeed, fusing the Fc domain of human IgG1 or mouse IgG2a either to allergens (Saxon et al, 2008) or worm Ags (Mekhaiel et al, 2011b) can significantly inhibit immune responses, and one study has demonstrated specific humoral tolerance with certain types of Ags and not others (Logan et al, 2009). Therefore, the choice of Ag incorporated into monomeric Fc-fusion based vaccines should most likely include Ags recognized by innate receptors on APCs.

Yet a recent study with malarial Ags also showed that the fused Ag can also affect Fc-fusion-FcγR interactions (Mekhaiel et al, 2011b). In this work, fusions to different malaria Ags showed markedly different affinities for FcγRIIA^H131^ and FcγRIIIA/B, while their interaction with FcRn, FcγRI and FcγRIIA^R131^ was not significantly altered. These differences between FcRn and the two FcγRs is most likely due to the different interaction sites on IgG: the binding site for FcRn on IgG is within the CH2–CH3 junction, whereas the site for FcγRs localizes to the lower hinge-region of the CH2 domain (Woof & Burton, 2004; Fig 1B). However the binding differences seen between FcγRs most likely occurs due to particular properties of the Ags in the area of the lower hinge region and to discrimination by the different FcγRs receptors for these properties (Mekhaiel et al, 2011b). These findings provide a useful background for future work and suggests that the hinge region could be exploited to produce Fc-fusion proteins with even greater specificity in receptor activation, as witnessed by the subtle diversification of this region over long periods of evolution (Redpath et al, 1998). The significant impact of different Ags on binding to FcγRs and FcRn, independent of their Fc architecture, emphasizes the importance of considering both subtle and gross structural changes imparted by different Ags in each individual Fc-fusion.

## Glycosylation of Fc-fusion proteins and their use in IVIG

Human IgGs share a conserved carbohydrate attached at asparagine 297 (Asn297) within their Fc-moieties (Fig 1A). Sugars anchored to Asn297 share a biantennary configuration of four *N*-acetyl-glucosamine (GlcNAc) and three mannose residues, with changeable numbers of galactose, fucose and sialic acid residues essential for biological functioning of the IgG-Fc (Jefferis, 2009). Fc-glycosylation is required for therapeutic Abs and Fc-fusions to elicit their effector functions and this is the main reason for producing these proteins in eukaryotic expression systems (Jefferis, 2009). Fc-fusion proteins that express glycoforms with improved binding for FcRs or complement receptors found on APCs may make more effective vaccines. Most studies investigating the contribution of specific sugars to the therapeutic efficacy of IgG1 have been studied in the context of FcγRIIIA-mediated ADCC and CDC. Since professional APCs, including DCs mostly express FcγRIIA/FcγRIIB (and not FcγRIIIA), the impact of individual sugars to Ag processing and presentation by Fc-fusions remains to be thoroughly investigated.

No clearer example exists for the importance of Asn297 to the function of the Fc than the recent demonstration that sialic acid enriched Fc-fragments are responsible for the beneficial effect of intravenous immunoglobulin (IVIG) therapy in preventing autoimmune disease (Anthony et al, 2011). These sialic acid residues interact with inhibitory receptors, including DC-specific intercellular adhesion molecule-3-grabbing non-integrin (Anthony et al, 2011; Schwab et al, 2012) and CD22 (Siglec-2) on B cells (Seite et al, 2010), that mediate the suppressive action of IVIG. Since less than 5% of IgG antibodies found in commercial preparations of IVIG are sialylated, extremely high doses of IVIG need to be administered to patients (2 g/kg bodyweight), and this can lead to significant adverse events in some people. Strategies that increase the sialic acid content of the Fc may therefore significantly improve the therapeutic capacity of IVIG or recombinant Fc-fragments.

One of the best studied sialic acid interactions is the influenza virus trimeric haemagglutinin, which can achieve affinities up to 10^8^ M^−1^ compared with around 4 × 10^2^ M^−1^ when one or both units are not in a multivalent state (Mammen et al, 1998). Hence, as an increase in avidity can enhance an inherently weak substrate affinity, presenting terminal sialic acid residues on a hexameric-Fc scaffold would likewise be expected to significantly enhance receptor binding, and thus may have potential for treating autoimmune disease (Fig 2A). That this is the case has been shown with nitrophenol-specific Abs recognizing NeuAc coupled to nitrophenol that efficiently assembled IgM-CD22 complexes on the surface of naive B cells (Adachi et al, 2011; O'Reilly et al, 2008). Not surprisingly IgM as a decavalent protein scaffold was more effective than either IgA or IgG, a finding supported by observations that IgM-enriched IVIG is more effective than IgG in controlling autoimmune disease (Kaveri et al, 2012). These proof-of-concept studies suggest that targeting of Siglec receptors by hexameric Fc-scaffolds can indeed be exploited for therapeutic applications (Mekhaiel et al, 2011b).

The anti-inflammatory properties of the sialylated Fc suggest that Fc-fusion proteins lacking sialic acid (or other sugars) should make better vaccines. That glycosylation status determines the pro- or anti-inflammatory ability of IgG is consistent with the observation that decreased sialylation and galactosylation of IgG are observed in patients with arthritis (Jefferis, 2009), and modifications to the carbohydrate content of IgG have been posited as a molecular explanation for the ‘hygiene-hypothesis’ (Mekhaiel et al, 2011a). The important contribution of specific sugars is exemplified by de-fucosylated therapeutic Abs that exhibit increased ADCC activity (Jefferis, 2009; Peipp et al, 2008; Shoji-Hosaka et al, 2006). Since macrophages and DCs also express a receptor for mannose and GlcNAc residues, fully galactosylated Fc-fusions may undergo minimal uptake by DCs (Carrillo-Conde et al, 2011).

## Fc-fusion proteins in non-clinical applications

The increased experience with Fc-fusions as therapeutic agents has increased confidence in the Fc-fusion construct more generally as a reliable and versatile platform for routine use in non-clinical applications, such as flow cytometry, immunohistochemistry and protein microarray devices (Flanagan et al, 2007). In these, the Fc region serves primarily as a well-behaved (stably and independently folding) module to which many different proteins can be attached and remain functional. In fact, linkage to the Fc domain can confer greater stability to some proteins, which has been recently exploited to enhance the expression of proteins in mammalian cells that are otherwise difficult to produce (Zhang et al, 2010). Still, based on the aforementioned results with fused Ags, it is likely that some proteins will not be functional when attached to the Fc-IgG1 domain. The Fc-IgG3, with its longer hinge region, should probably be investigated as a more universally effective platform for these types of applications.

**Pending issues**

Determine if polymeric versions of Fc and Fc-fusions are more potent, and thereby less expensive as replacements for IVIG or monomeric Fc-fusions in the treatment of human disease, as well as capable of entirely different functionalities depending on the Ig class used in the construct. For example, design monomeric or polymeric Fc-fusions with selective binding to FcRs, FcRn or FcRLs.

Use of synthetic biology approaches to design more efficient polymerizing tags that allow improved self-assembly and functionality of the Fc, *e.g.* the use of coiled-coils. Synthetic biology has the power to transform improvements to the antibody molecule (http://www.bris.ac.uk/scn/syntheticbiology).

Better understand the role of glycosylation and sialylation in the function of both monomeric and polymeric Fc and Fc-fusions.

Determine the critical Fc- and glycan-receptors involved in binding monomeric and polymeric Fc-fusions. For example, explore the contribution of recently discovered receptors (*e.g.* FcRL5, FcRLA and TRIM21) to functioning of monomeric and/or polymeric Fc and Fc-fusion proteins.

Speed the transition of human Fc-fusions to the clinic by making improved humanized Fc mouse models.

Determine if Fc-fusions to Ags make useful vaccines for diseases other than those caused by viruses, and whether polymeric versions are even more potent by nature of their increased avidity binding.

One advantage of Fc-fusions for protein microarray applications is the identical means by which the many different linked proteins can be simultaneously attached to a solid surface, typically via interactions between the Fc-domain and surface-bound protein G/A. Recently this has been further exploited in high throughput approaches to identify novel low affinity protein interactions (Gonzalez et al, 2005; Hsu et al, 2009; Lin et al, 2003; Ramani et al, 2012; Wojtowicz et al, 2007; Wright, 2009). Although mass spectrometry and yeast-two-hybrid technologies have proved to be very useful for investigating protein-protein interactions, they are hampered by their inability to detect low affinity binding events that are often observed during cell–cell or cell–matrix contact (Wright, 2009). To enhance detection of low-affinity interactions, Fc-fusion proteins have been assembled into multivalent complexes using scaffolds such as protein-G/A microbeads (Fig 2C). A similar assembly of receptors could be envisioned using the hexameric-Fc platform discussed above (Fig 2C; Mekhaiel et al, 2011b). Rather than requiring an additional step of pre-assembling the Fc onto protein-A beads, these more natural scaffolds could be used to pull out low affinity interactions in situations where the presence of large beads may not be optimal (such as sensitive cell cultures or *in vivo*), or where the presence of protein G/A may result in the identification of false-positive interactions. Furthermore, strategies involving scaffolds (such as protein-A beads or ELISA plates) have the additional disadvantage of not being able to ensure equivalent immobilization levels of Fc-fusion to the scaffold, which is known to impact the cut off thresholds for detecting genuine interactions. By cloning ‘bait’ proteins into hexameric IgG1-Fc, and ‘prey’ proteins into the hexameric IgM-Fc, it may be possible to investigate interactions where both ‘bait’ and ‘prey’ must be multivalent for any interaction to occur. Such engineering of the Fc for increased avidity binding may also lead to the development of more sensitive diagnostic assays that are scaffold independent.

Naturally, the use of Fc-fusions as research tools when studying immune cells or extracellular proteins thereof is complicated by the ability of the Fc-domain itself to bind FcRs, as this would cause unwanted positive binding signals or biological effects. Thus hexameric Fc-fusions that no longer engage FcRs or complement due to the presence of the fusion partner or defined mutagenesis may be ideal, especially if they can be combined with mass spectrometry approaches for high throughput identification. The successful use of synthetic biology to develop entirely man-made Ag-binding sites that improve on nature may also now be applied to the Fc to generate more effective tools and therapies (Sidhu & Fellouse, 2006).

## Concluding remarks

Fc-fusions are now a well-established class of therapeutics. As the biological processes consequent to effector activation become better understood, it is likely that more precise targeting of effector systems will characterize future developments in this direction (Supporting Information Table S2). Yet their present success demonstrates a fundamental effectiveness of this type of medication that portends well for their application in vaccines. The discovery that mucosal delivery of Fc-fusions to Ags can protect mice from several viruses via FcRn provides urgent incentive to determine if such strategies are translatable to humans and non-viral pathogens, such as malaria and tuberculosis. To this end, the continued improvement and humanization of animal models to recapitulate human FcR structure and functional diversity will allow speedier evaluation and translation of these reagents to the clinic (Smith et al, 2012). Whether different mechanisms of action via FcγRs, or with different Fc-scaffolds or polymeric fusions, are similarly effective likewise deserves closer scrutiny. How Fc-fusions can be delivered without the requirement for chemical adjuvants is also a significant technical challenge. Work along these directions will undoubtedly reveal as much about the biological processes as about the effects of the Fc-fusions themselves, and further validate the Fc-fusion construct as a generally useful research platform for both clinical and non-clinical applications.

## For more information

http://www.drugbank.ca/

The DrugBank database is a unique bioinformatics and chemoinformatics resource that combines detailed drug (*i.e.* chemical, pharmacological and pharmaceutical) data with comprehensive drug target (*i.e.* sequence, structure and pathway) information.

http://clinicaltrials.gov/

Searchable database providing patients, family members and the public with information about current ongoing clinical research studies.

http://www.fda.gov/

The US Food and Drug Administration provides useful information and databases on drug approvals, drug safety and recall and alerts.

http://www.lamerie.com/

The consultancy business of La Merie Business Intelligence was established in 2002 by Ulrich Martin, MD, based on his extensive experience in the biopharmaceutical industry both as a physician specializing in pharmacology and as a co-founder and CEO of a product development biotech company. La Merie Business Intelligence started the online presence of its biotech portal PipelineReview.com in 2006 as an R&D news provider and online shop for reports and subscription services. PipelineReview.com is owned and operated by La Merie SL. All reports and contents are produced by La Merie Publishing. In addition to publication of reports, La Merie Publishing prepares on demand, customized reports for its clients.

http://www.linkedin.com/groups/IMMUNOTHERAPY-2147790

Antibody engineering and design forum, antibody network, and immunotherapy groups can be found at LinkedIn and provide a useful repository of advice and topical issues in the field.

http://www.landesbioscience.com/journals/mabs/

The journal mAbs provides a forum for communication on all topics relevant to antibody research and development, with a focus on therapeutics.

## Supplementary material

Detailed facts of importance to specialist readers are published as ”Supporting Information”. Such documents are peer-reviewed, but not copy-edited or typeset. They are made available as submitted by the authors.

## References

[b1] Adachi T, Harumiya S, Takematsu H, Kozutsumi Y, Wabl M, Fujimoto M, Tedder TF (2011). CD22 serves as a receptor for soluble IgM. Eur J Immunol.

[b2] Ammann JU, Jahnke M, Dyson MR, Kaufman J, Trowsdale J (2012). Detection of weak receptor-ligand interactions using IgM and J-chain-based fusion proteins. Eur J Immunol.

[b3] Anthony RM, Kobayashi T, Wermeling F, Ravetch JV (2011). Intravenous gammaglobulin suppresses inflammation through a novel T(H)2 pathway. Nature.

[b4] Arnason JE, Campigotto F, Neuberg D, Bussel JB (2012). Abnormalities in IgA and IgM are associated with treatment-resistant ITP. Blood.

[b5] Bajtay Z, Csomor E, Sandor N, Erdei A (2006). Expression and role of Fc- and complement-receptors on human dendritic cells. Immunol Lett.

[b6] Bakema JE, van Egmond M (2011a). The human immunoglobulin A Fc receptor FcalphaRI: a multifaceted regulator of mucosal immunity. Mucosal Immunol.

[b7] Bakema JE, van Egmond M (2011b). Immunoglobulin A: a next generation of therapeutic antibodies. MAbs.

[b8] Bakema JE, Ganzevles SH, Fluitsma DM, Schilham MW, Beelen RH, Valerius T, Lohse S, Glennie MJ, Medema JP, van Egmond M (2011). Targeting FcalphaRI on polymorphonuclear cells induces tumor cell killing through autophagy. J Immunol.

[b9] Baker K, Qiao SW, Kuo TT, Aveson VG, Platzer B, Andersen JT, Sandlie I, Chen Z, de Haar C, Lencer WI (2011). Neonatal Fc receptor for IgG (FcRn) regulates cross-presentation of IgG immune complexes by CD8-CD11b+ dendritic cells. Proc Natl Acad Sci USA.

[b10] Balu S, Reljic R, Lewis MJ, Pleass RJ, McIntosh R, van Kooten C, van Egmond M, Challacombe S, Woof JM, Ivanyi J (2011). A novel human IgA monoclonal antibody protects against tuberculosis. J Immunol.

[b11] Beck A, Reichert JM (2011). Therapeutic Fc-fusion proteins and peptides as successful alternatives to antibodies. MAbs.

[b12] Biburger M, Aschermann S, Schwab I, Lux A, Albert H, Danzer H, Woigk M, Dudziak D, Nimmerjahn F (2011). Monocyte subsets responsible for immunoglobulin G-dependent effector functions in vivo. Immunity.

[b13] Bitonti AJ, Dumont JA, Low SC, Peters RT, Kropp KE, Palombella VJ, Stattel JM, Lu Y, Tan CA, Song JJ (2004). Pulmonary delivery of an erythropoietin Fc fusion protein in non-human primates through an immunoglobulin transport pathway. Proc Natl Acad Sci USA.

[b14] Bruhns P, Iannascoli B, England P, Mancardi DA, Fernandez N, Jorieux S, Daeron M (2009). Specificity and affinity of human Fcgamma receptors and their polymorphic variants for human IgG subclasses. Blood.

[b15] Canal F, Sanchis J, Vicent MJ (2011). Polymer–drug conjugates as nano-sized medicines. Curr Opin Biotechnol.

[b16] Capon DJ, Chamow SM, Mordenti J, Marsters SA, Gregory T, Mitsuya H, Byrn RA, Lucas C, Wurm FM, Groopman JE (1989). Designing CD4 immunoadhesins for AIDS therapy. Nature.

[b17] Carrillo-Conde B, Song EH, Chavez-Santoscoy A, Phanse Y, Ramer-Tait AE, Pohl NL, Wannemuehler MJ, Bellaire BH, Narasimhan B (2011). Mannose-functionalized “pathogen-like” polyanhydride nanoparticles target C-type lectin receptors on dendritic cells. Mol Pharm.

[b18] Carter PJ (2011). Introduction to current and future protein therapeutics: a protein engineering perspective. Exp Cell Res.

[b19] Cervenak J, Bender B, Schneider Z, Magna M, Carstea BV, Liliom K, Erdei A, Bosze Z, Kacskovics I (2011). Neonatal FcR overexpression boosts humoral immune response in transgenic mice. J Immunol.

[b20] Congy-Jolivet N, Probst A, Watier H, Thibault G (2007). Recombinant therapeutic monoclonal antibodies: mechanisms of action in relation to structural and functional duality. Crit Rev Oncol/Hematol.

[b21] Cox GN, Smith DJ, Carlson SJ, Bendele AM, Chlipala EA, Doherty DH (2004). Enhanced circulating half-life and hematopoietic properties of a human granulocyte colony-stimulating factor/immunoglobulin fusion protein. Exp Hematol.

[b22] Czajkowsky DM, Salanti A, Ditlev SB, Shao Z, Ghumra A, Rowe JA, Pleass RJ (2010). IgM, Fc mu Rs, and malarial immune evasion. J Immunol.

[b23] Daeron M, Malbec O, Latour S, Arock M, Fridman WH (1995). Regulation of high-affinity IgE receptor-mediated mast cell activation by murine low-affinity IgG receptors. J Clin Invest.

[b24] Dall'Acqua WF, Kiener PA, Wu H (2006). Properties of human IgG1s engineered for enhanced binding to the neonatal Fc receptor (FcRn). J Biol Chem.

[b25] Davis JH, Aperlo C, Li Y, Kurosawa E, Lan Y, Lo KM, Huston JS (2010). SEEDbodies: fusion proteins based on strand-exchange engineered domain (SEED) CH3 heterodimers in an Fc analogue platform for asymmetric binders or immunofusions and bispecific antibodies. Protein Eng Des Sel.

[b26] Flanagan ML, Arias RS, Hu P, Khawli LA, Epstein AL (2007). Soluble Fc fusion proteins for biomedical research. Methods Mol Biol.

[b27] Gaboriaud C, Juanhuix J, Gruez A, Lacroix M, Darnault C, Pignol D, Verger D, Fontecilla-Camps JC, Arlaud GJ (2003). The crystal structure of the globular head of complement protein C1q provides a basis for its versatile recognition properties. J Biol Chem.

[b28] Getahun A, Heyman B (2006). How antibodies act as natural adjuvants. Immunol Lett.

[b29] Getahun A, Dahlstrom J, Wernersson S, Heyman B (2004). IgG2a-mediated enhancement of antibody and T cell responses and its relation to inhibitory and activating Fc gamma receptors. J Immunol.

[b30] Gil M, Bieniasz M, Wierzbicki A, Bambach BJ, Rokita H, Kozbor D (2009). Targeting a mimotope vaccine to activating Fcgamma receptors empowers dendritic cells to prime specific CD8+ T cell responses in tumor-bearing mice. J Immunol.

[b31] Gonzalez LC, Loyet KM, Calemine-Fenaux J, Chauhan V, Wranik B, Ouyang W, Eaton DL (2005). A coreceptor interaction between the CD28 and TNF receptor family members B and T lymphocyte attenuator and herpesvirus entry mediator. Proc Natl Acad Sci USA.

[b32] Griffiss JM, Goroff DK (1983). IgA blocks IgM and IgG-initiated immune lysis by separate molecular mechanisms. J Immunol.

[b33] Harte PG, Cooke A, Playfair JH (1983). Specific monoclonal IgM is a potent adjuvant in murine malaria vaccination. Nature.

[b34] Haynes BF, Gilbert PB, McElrath MJ, Zolla-Pazner S, Tomaras GD, Alam SM, Evans DT, Montefiori DC, Karnasuta C, Sutthent R (2012). Immune-correlates analysis of an HIV-1 vaccine efficacy trial. N Engl J Med.

[b35] Heldwein EE, Krummenacher C (2008). Entry of herpesviruses into mammalian cells. Cell Mol Life Sci.

[b36] Hoffman HM, Throne ML, Amar NJ, Sebai M, Kivitz AJ, Kavanaugh A, Weinstein SP, Belomestnov P, Yancopoulos GD, Stahl N (2008). Efficacy and safety of rilonacept (interleukin-1 Trap) in patients with cryopyrin-associated periodic syndromes: results from two sequential placebo-controlled studies. Arthritis Rheum.

[b37] Horiuchi T, Mitoma H, Harashima S, Tsukamoto H, Shimoda T (2010). Transmembrane TNF-alpha: structure, function and interaction with anti-TNF agents. Rheumatology (Oxford).

[b38] Hsu TL, Cheng SC, Yang WB, Chin SW, Chen BH, Huang MT, Hsieh SL, Wong CH (2009). Profiling carbohydrate-receptor interaction with recombinant innate immunity receptor-Fc fusion proteins. J Biol Chem.

[b39] Huang C (2009). Receptor-Fc fusion therapeutics, traps, and MIMETIBODY technology. Curr Opin Biotechnol.

[b40] Idusogie EE, Wong PY, Presta LG, Gazzano-Santoro H, Totpal K, Ultsch M, Mulkerrin MG (2001). Engineered antibodies with increased activity to recruit complement. J Immunol.

[b41] Jefferis R (2007). Antibody therapeutics: isotype and glycoform selection. Expert Opin Biol Ther.

[b42] Jefferis R (2009). Glycosylation as a strategy to improve antibody-based therapeutics. Nat Rev Drug Discov.

[b43] Jensen MA, Arnason BG, White DM (2007). A novel Fc gamma receptor ligand augments humoral responses by targeting antigen to Fc gamma receptors. Eur J Immunol.

[b44] Jung K, Lee JE, Kim HZ, Kim HM, Park BS, Hwang SI, Lee JO, Kim SC, Koh GY (2009). Toll-like receptor 4 decoy, TOY, attenuates gram-negative bacterial sepsis. PloS one.

[b45] Karagiannis SN, Josephs DH, Karagiannis P, Gilbert AE, Saul L, Rudman SM, Dodev T, Koers A, Blower PJ, Corrigan C (2011). Recombinant IgE antibodies for passive immunotherapy of solid tumours: from concept towards clinical application. Cancer Immunol Immunother.

[b46] Kardava L, Moir S, Wang W, Ho J, Buckner CM, Posada JG, O'Shea MA, Roby G, Chen J, Sohn HW (2011). Attenuation of HIV-associated human B cell exhaustion by siRNA downregulation of inhibitory receptors. J Clin Invest.

[b47] Kaveri SV, Silverman GJ, Bayry J (2012). Natural IgM in immune equilibrium and harnessing their therapeutic potential. J Immunol.

[b48] Kontermann RE (2011). Strategies for extended serum half-life of protein therapeutics. Curr Opin Biotechnol.

[b49] Kumar M, Hunag Y, Glinka Y, Prud'homme GJ, Wang Q (2007). Gene therapy of diabetes using a novel GLP-1/IgG1-Fc fusion construct normalizes glucose levels in db/db mice. Gene Ther.

[b50] Leroux-Roels G (2010). Unmet needs in modern vaccinology: adjuvants to improve the immune response. Vaccine.

[b51] Li F, Ravetch JV (2011). Inhibitory Fcgamma receptor engagement drives adjuvant and anti-tumor activities of agonistic CD40 antibodies. Science.

[b52] Lin JC, Ho WH, Gurney A, Rosenthal A (2003). The netrin-G1 ligand NGL-1 promotes the outgrowth of thalamocortical axons. Nat Neurosci.

[b53] Lofblom J, Frejd FY, Stahl S (2011). Non-immunoglobulin based protein scaffolds. Curr Opin Biotechnol.

[b54] Logan GJ, Wang L, Zheng M, Ginn SL, Coppel RL, Alexander IE (2009). Antigen-specific humoral tolerance or immune augmentation induced by intramuscular delivery of adeno-associated viruses encoding CTLA4-Ig-antigen fusion molecules. Gene Ther.

[b55] Lohse S, Derer S, Beyer T, Klausz K, Peipp M, Leusen JH, van de Winkel JG, Dechant M, Valerius T (2011). Recombinant dimeric IgA antibodies against the epidermal growth factor receptor mediate effective tumor cell killing. J Immunol.

[b56] Lu L, Palaniyandi S, Zeng R, Bai Y, Liu X, Wang Y, Pauza CD, Roopenian DC, Zhu X (2011). A neonatal Fc receptor-targeted mucosal vaccine strategy effectively induces HIV-1 antigen-specific immunity to genital infection. J Virol.

[b57] Mammen M, Choi SK, Whitesides GM (1998). Polyvalent interactions in biological systems: implications for design and use of multivalent ligands and inhibitors. Angew Chem Int Edit.

[b58] Martin WL, Bjorkman PJ (1999). Characterization of the 2:1 complex between the class I MHC-related Fc receptor and its Fc ligand in solution. Biochemistry.

[b59] Mathew GD, Qualtiere LF, Neel HB, Pearson GR (1981). IgA antibody, antibody-dependent cellular cytotoxicity and prognosis in patients with nasopharyngeal carcinoma. Int J Cancer J Int Cancer.

[b60] McEwan WA, Mallery DL, Rhodes DA, Trowsdale J, James LC (2011). Intracellular antibody-mediated immunity and the role of TRIM21. BioEssays.

[b61] Mekhaiel DN, Daniel-Ribeiro CT, Cooper PJ, Pleass RJ (2011a). Do regulatory antibodies offer an alternative mechanism to explain the hygiene hypothesis. Trends Parasitol.

[b62] Mekhaiel DN, Czajkowsky DM, Andersen JT, Shi J, El-Faham M, Doenhoff M, McIntosh RS, Sandlie I, He J, Hu J (2011b). Polymeric human Fc-fusion proteins with modified effector functions. Sci Rep.

[b63] Morell A, Terry WD, Waldmann TA (1970). Metabolic properties of IgG subclasses in man. J Clin Invest.

[b64] Muda M, Gross AW, Dawson JP, He C, Kurosawa E, Schweickhardt R, Dugas M, Soloviev M, Bernhardt A, Fischer D (2011). Therapeutic assessment of SEED: a new engineered antibody platform designed to generate mono- and bispecific antibodies. Protein Eng Des Sel.

[b65] Nagashima H, Kaneko K, Yamanoi A, Motoi S, Konakahara S, Kohroki J, Masuho Y (2011). TNF receptor II fusion protein with tandemly repeated Fc domains. J Biochem.

[b66] Nelson AL (2010). Antibody fragments: hope and hype. MAbs.

[b67] Nelson AL, Reichert JM (2009). Development trends for therapeutic antibody fragments. Nat Biotechnol.

[b68] Nigro EA, Soprana E, Brini AT, Ambrosi A, Yenagi VA, Dombrowicz D, Siccardi AG, Vangelista L (2012). An antitumor cellular vaccine based on a mini-membrane IgE. J Immunol.

[b69] Nimmerjahn F, Ravetch JV (2008). Fcgamma receptors as regulators of immune responses. Nat Rev Immunol.

[b70] O'Reilly MK, Collins BE, Han S, Liao L, Rillahan C, Kitov PI, Bundle DR, Paulson JC (2008). Bifunctional CD22 ligands use multimeric immunoglobulins as protein scaffolds in assembly of immune complexes on B cells. J Am Chem Soc.

[b71] Peipp M, Dechant M, Valerius T (2008). Effector mechanisms of therapeutic antibodies against ErbB receptors. Curr Opin Immunol.

[b72] Peppel K, Crawford D, Beutler B (1991). A tumor necrosis factor (TNF) receptor-IgG heavy chain chimeric protein as a bivalent antagonist of TNF activity. J Exp Med.

[b73] Pfirsch-Maisonnas S, Aloulou M, Xu T, Claver J, Kanamaru Y, Tiwari M, Launay P, Monteiro RC, Blank U (2011). Inhibitory ITAM signaling traps activating receptors with the phosphatase SHP-1 to form polarized “inhibisome” clusters. Sci Signal.

[b74] Quan CP, Watanabe S, Forestier F, Bouvet JP (1998). Human amniotic IgA inhibits natural IgG autoantibodies of maternal or unrelated origin. Eur J Immunol.

[b75] Ramani SR, Tom I, Lewin-Koh N, Wranik B, Depalatis L, Zhang J, Eaton D, Gonzalez LC (2012). A secreted protein microarray platform for extracellular protein interaction discovery. Anal Biochem.

[b76] Rapaka RR, Ricks DM, Alcorn JF, Chen K, Khader SA, Zheng M, Plevy S, Bengten E, Kolls JK (2010). Conserved natural IgM antibodies mediate innate and adaptive immunity against the opportunistic fungus *Pneumocystis murina*. J Exp Med.

[b77] Reali E, Greiner JW, Corti A, Gould HJ, Bottazzoli F, Paganelli G, Schlom J, Siccardi AG (2001). IgEs targeted on tumor cells: therapeutic activity and potential in the design of tumor vaccines. Cancer Res.

[b78] Redpath S, Michaelsen TE, Sandlie I, Clark MR (1998). The influence of the hinge region length in binding of human IgG to human Fcgamma receptors. Hum Immunol.

[b79] Regnault A, Lankar D, Lacabanne V, Rodriguez A, Thery C, Rescigno M, Saito T, Verbeek S, Bonnerot C, Ricciardi-Castagnoli P (1999). Fcgamma receptor-mediated induction of dendritic cell maturation and major histocompatibility complex class I-restricted antigen presentation after immune complex internalization. J Exp Med.

[b80] Reichert JM (2011). Antibody-based therapeutics to watch in 2011. MAbs.

[b81] Roopenian DC, Akilesh S (2007). FcRn: the neonatal Fc receptor comes of age. Nat Rev Immunol.

[b82] Rossi EA, Goldenberg DM, Cardillo TM, Stein R, Chang CH (2009). Hexavalent bispecific antibodies represent a new class of anticancer therapeutics: 1. Properties of anti-CD20/CD22 antibodies in lymphoma. Blood.

[b83] Saad JS, Miller J, Tai J, Kim A, Ghanam RH, Summers MF (2006). Structural basis for targeting HIV-1 Gag proteins to the plasma membrane for virus assembly. Proc Natl Acad Sci USA.

[b84] Salfeld JG (2007). Isotype selection in antibody engineering. Nat Biotechnol.

[b85] Sandborn WJ, Hanauer SB, Katz S, Safdi M, Wolf DG, Baerg RD, Tremaine WJ, Johnson T, Diehl NN, Zinsmeister AR (2001). Etanercept for active Crohn's disease: a randomized, double-blind, placebo-controlled trial. Gastroenterology.

[b86] Santiago T, Kulemzin SV, Reshetnikova ES, Chikaev NA, Volkova OY, Mechetina LV, Zhao M, Davis RS, Taranin AV, Najakshin AM (2011). FCRLA is a resident endoplasmic reticulum protein that associates with intracellular Igs, IgM, IgG and IgA. Int Immunol.

[b87] Saxon A, Kepley C, Zhang K (2008). “Accentuate the negative, eliminate the positive”: engineering allergy therapeutics to block allergic reactivity through negative signaling. J Allergy Clin Immunol.

[b88] Scallon B, Cai A, Solowski N, Rosenberg A, Song XY, Shealy D, Wagner C (2002). Binding and functional comparisons of two types of tumor necrosis factor antagonists. J Pharmacol Exp Ther.

[b89] Schwab I, Biburger M, Kronke G, Schett G, Nimmerjahn F (2012). IVIg-mediated amelioration of ITP in mice is dependent on sialic acid and SIGNR1. Eur J Immunol.

[b90] Seite JF, Cornec D, Renaudineau Y, Youinou P, Mageed RA, Hillion S (2010). IVIg modulates BCR signaling through CD22 and promotes apoptosis in mature human B lymphocytes. Blood.

[b91] Shi J, McIntosh RS, Adame-Gallegos J, Dehal PK, van Egmond M, van de Winkel J, Draper SJ, Forbes EK, Corran PH, Holder AA (2011). The generation and evaluation of recombinant human IgA specific for *Plasmodium falciparum* merozoite surface protein 1-19 (PfMSP1 19). BMC Biotechnol.

[b92] Shinkawa T, Nakamura K, Yamane N, Shoji-Hosaka E, Kanda Y, Sakurada M, Uchida K, Anazawa H, Satoh M, Yamasaki M (2003). The absence of fucose but not the presence of galactose or bisecting *N**N*-acetylglucosamine of human IgG1 complex-type oligosaccharides shows the critical role of enhancing antibody-dependent cellular cytotoxicity. J Biol Chem.

[b93] Shoji-Hosaka E, Kobayashi Y, Wakitani M, Uchida K, Niwa R, Nakamura K, Shitara K (2006). Enhanced Fc-dependent cellular cytotoxicity of Fc fusion proteins derived from TNF receptor II and LFA-3 by fucose removal from Asn-linked oligosaccharides. J Biochem.

[b94] Sidhu SS, Fellouse FA (2006). Synthetic therapeutic antibodies. Nat Chem Biol.

[b95] Smith P, Dilillo DJ, Bournazos S, Li F, Ravetch JV (2012). Mouse model recapitulating human Fcgamma receptor structural and functional diversity. Proc Natl Acad Sci USA.

[b96] Soltani N, Kumar M, Glinka Y, Prud'homme GJ, Wang Q (2007). In vivo expression of GLP-1/IgG-Fc fusion protein enhances beta-cell mass and protects against streptozotocin-induced diabetes. Gene Ther.

[b97] Stager S, Alexander J, Kirby AC, Botto M, Rooijen NV, Smith DF, Brombacher F, Kaye PM (2003). Natural antibodies and complement are endogenous adjuvants for vaccine-induced CD8+ T-cell responses. Nat Med.

[b98] Stapleton NM, Andersen JT, Stemerding AM, Bjarnarson SP, Verheul RC, Gerritsen J, Zhao Y, Kleijer M, Sandlie I, de Haas M (2011). Competition for FcRn-mediated transport gives rise to short half-life of human IgG3 and offers therapeutic potential. Nat Commun.

[b99] Stavenhagen JB, Gorlatov S, Tuaillon N, Rankin CT, Li H, Burke S, Huang L, Johnson S, Koenig S, Bonvini E (2008). Enhancing the potency of therapeutic monoclonal antibodies via Fc optimization. Adv Enzyme Regul.

[b100] Strohl WR (2009). Optimization of Fc-mediated effector functions of monoclonal antibodies. Curr Opin Biotechnol.

[b101] Strohl WR, Knight DM (2009). Discovery and development of biopharmaceuticals: current issues. Curr Opin Biotechnol.

[b102] Suzuki T, Ishii-Watabe A, Tada M, Kobayashi T, Kanayasu-Toyoda T, Kawanishi T, Yamaguchi T (2010). Importance of neonatal FcR in regulating the serum half-life of therapeutic proteins containing the Fc domain of human IgG1: a comparative study of the affinity of monoclonal antibodies and Fc-fusion proteins to human neonatal FcR. J Immunol.

[b103] Treon SP, Mitsiades C, Mitsiades N, Young G, Doss D, Schlossman R, Anderson KC (2001). Tumor cell expression of CD59 is associated with resistance to CD20 serotherapy in patients with B-cell malignancies. J Immunother.

[b104] Van den Brande JM, Braat H, van den Brink GR, Versteeg HH, Bauer CA, Hoedemaeker I, van Montfrans C, Hommes DW, Peppelenbosch MP, van Deventer SJ (2003). Infliximab but not etanercept induces apoptosis in lamina propria T-lymphocytes from patients with Crohn's disease. Gastroenterology.

[b105] Van Scott MR, Mertsching E, Negrou E, Miles J, Stallings HW, Graff C, Kehry MR (2008). Systemic administration of an Fcgamma-Fc(epsilon)-fusion protein in house dust mite sensitive nonhuman primates. Clin Immunol.

[b106] Veeramani S, Wang SY, Dahle C, Blackwell S, Jacobus L, Knutson T, Button A, Link BK, Weiner GJ (2011). Rituximab infusion induces NK activation in lymphoma patients with the high-affinity CD16 polymorphism. Blood.

[b107] Vire B, David A, Wiestner A (2011). TOSO, the Fcmicro receptor, is highly expressed on chronic lymphocytic leukemia B cells, internalizes upon IgM binding, shuttles to the lysosome, and is downregulated in response to TLR activation. J Immunol.

[b108] Vollmers HP, Brandlein S (2006). Natural IgM antibodies: the orphaned molecules in immune surveillance. Adv Drug Deliv Rev.

[b109] Wang Q, Chen K, Liu R, Zhao F, Gupta S, Zhang N, Prud'homme GJ (2010). Novel GLP-1 fusion chimera as potent long acting GLP-1 receptor agonist. PloS one.

[b110] Weiner GJ (2010). Rituximab: mechanism of action. Sem Hematol.

[b111] West AP, Galimidi RP, Gnanapragasam PN, Bjorkman PJ (2012). Single-chain Fv-based anti-HIV proteins: potential and limitations. J Virol.

[b112] Wilson TJ, Gilfillan S, Colonna M (2010). Fc receptor-like A associates with intracellular IgG and IgM but is dispensable for antigen-specific immune responses. J Immunol.

[b113] Wilson TJ, Fuchs A, Colonna M (2012). Cutting edge: human FcRL4 and FcRL5 are receptors for IgA and IgG. J Immunol.

[b114] Wines BD, Trist HM, Farrugia W, Ngo C, Trowsdale J, Areschoug T, Lindahl G, Fraser JD, Ramsland PA (2012). A conserved host and pathogen recognition site on immunoglobulins: structural and functional aspects. Adv Exp Med Biol.

[b115] Wojtowicz WM, Wu W, Andre I, Qian B, Baker D, Zipursky SL (2007). A vast repertoire of Dscam binding specificities arises from modular interactions of variable Ig domains. Cell.

[b116] Woof JM, Burton DR (2004). Human antibody-Fc receptor interactions illuminated by crystal structures. Nat Rev Immunol.

[b117] Wright GJ (2009). Signal initiation in biological systems: the properties and detection of transient extracellular protein interactions. Mol Biosyst.

[b118] Ye L, Zeng R, Bai Y, Roopenian DC, Zhu X (2011). Efficient mucosal vaccination mediated by the neonatal Fc receptor. Nat Biotechnol.

[b119] Yeung YA, Leabman MK, Marvin JS, Qiu J, Adams CW, Lien S, Starovasnik MA, Lowman HB (2009). Engineering human IgG1 affinity to human neonatal Fc receptor: impact of affinity improvement on pharmacokinetics in primates. J Immunol.

[b120] Zalevsky J, Chamberlain AK, Horton HM, Karki S, Leung IW, Sproule TJ, Lazar GA, Roopenian DC, Desjarlais JR (2010). Enhanced antibody half-life improves in vivo activity. Nat Biotechnol.

[b121] Zhang J, Carter J, Siu S, O'Neill JW, Gates AH, Delaney J, Mehlin C (2010). Fusion partners as a tool for the expression of difficult proteins in mammalian cells. Curr Pharm Biotechnol.

[b122] Zhu X, Meng G, Dickinson BL, Li X, Mizoguchi E, Miao L, Wang Y, Robert C, Wu B, Smith PD (2001). MHC class I-related neonatal Fc receptor for IgG is functionally expressed in monocytes, intestinal macrophages, and dendritic cells. J Immunol.

