# Supplementary material for: Fc-fusion proteins: new developments and future perspectives
Source: EMBO Mol Med. 2012 Jul 26;4(10):1015–28. doi: 10.1002/emmm.201201379 (PMC3491832; doi:10.1002/emmm.201201379)
Supplement: Supplementary file 1 [file emmm0004-1015-SD1.pdf]

Fc-fusion proteins: new developments and future perspectives

Daniel M Czajkowsky[1,2], Jun Hu[2], Zhifeng Shao[1], and Richard J Pleass[3*]

[1]Key Laboratory of Systems Biomedicine (Ministry of Education) & State Key Laboratory of Oncogenes & Related Genes, Shanghai Jiao Tong University, Shanghai, China; [2]Laboratory of Physical Biology, Shanghai Institute of Applied Physics, Chinese Academy of Sciences, Shanghai, China; [3]Liverpool School of Tropical Medicine, Pembroke Place, Liverpool, L3 5QA, UK.

Supporting information table of contents

Table S1. Fc-fusion approaches in vaccines - page 1.

Table S2. Summary of modifications - page 2.

Table S1. Fc-fusion approaches in vaccines

Target	Type of Fc-fusion	Type of vaccine (route admin)	Immunogenicity/ Tolerogenicity	Neutralization *in vitro*	Neutralization *in vivo*	Mechanism of action	Ref
Clostridium botulinum	BoNT-hIgG1 Fc x2	Protein +mlipidA + squalene (s.c.)	Immunogenic in mice, Fc-fusion superior to non-targeted BoNT	Yes	n.d.	FcγR dependent	1
HIV	p24-mIgG1 and IgG2a	Protein or DNA (i.p./i.m.)	Immunogenic in mice, mouse IgG2a superior to mouse IgG1	Yes	n.d.	FcγR dependent ADCC & CDC independent	2
	p24-mIgG2a	Protein + CpG (i.n.)	Immunogenic in mice	Yes	Yes, on vaginal challenge	FcRn dependent	3
	gp41-hIgG1	Protein + FCA (s.c.)	Immunogenic in rabbits	Yes, in human PBMC assay	n.d.	n.d.	4
	gp120-hIgG1	Protein (no adjuvant)	Immunogenic in mice (no benefit to oligomerization Fc)	n.d.	n.d.	Fc-dependent	5
	g24-hIgA1	Protein + alum (s.c.)	Poorly immunogenic	n.d.	n.d.	n.d.	6
Influenza	Hemagglutinin -mIgG2a	Protein (i.p.)	Immunogenic in mice	Yes	n.d.	n.d.	2
	Hemagglutinin -hIgG1	Protein (s.c.)	Immunogenic in mice	Yes	n.d.	n.d.	7
Ebola	Filovirus glycoprotein-hIgG1	Protein (i.p. + FCA)	Immunogenic, Ab titers 1:64,000	Yes, in plaque reduction assay	7/8 mice protected	Fc-dependent	8
Hepatitis	HBsAg-mIgG2a	Lentiviral footpad or DNA (i.m.)	Immunogenic	Yes, FcγR induction of CD4+/CD8+ T cell responses	Seroconversion in HBsAg low Tg mice	n.d.	9, 10
HSV-2	gD-modified mIgG2a	Protein + CpG (i.n.)	Immunogenic, high mIgG2a responses when co-administered with CpG	Yes	80% survival on vaginal challenge Long-lived memory cells generated	FcRn dependent	11
Avian influenza	Hemagglutin HA1-hIgG1	Protein +SAS (s.c.)	Immunogenic, clade transcendent IgG generated	Yes	Yes, reduced viral replication and lung damage	90-100% survival	12
Pseudorabies virus	Mouse transferrin receptor-mgG1	Inactivated virus	Immunogenic, high mIgG2a elicited	Yes	Yes	n.d.	13
Malaria	PfMSP119-mIgG2a or hIgG1	Protein ± alum (i.p.)	Immunogenic as monomer, high IgG1 responses (no benefit to oligomerization)	n.d.	No, to i.p. challenge with infected erythrocytes	n.d.	14
	CTLA4-hIgG1-Fc-PyMSP4/5	Adenovirus (i.m.)	Tolerogenic at high dose	n.d.	n.d.	n.d.	15
Schistosomiasis	CE-mIgG2a	Protein (i.p.)	Poorly immunogenic compared to CE-HIS in Alum and complete ablation of IgE responses	n.d.	No, to cercarial challenge	n.d.	14
Cancer	Cytokine-mIgG1	DNA-injection	Yes, improved T cell responses	Yes	n.d.	n.d.	16

			compared with non-Fc fusions				
Cancer (neuroblastoma)	Mimotope-mIgG2a	Protein	Yes, with DC's pulsed with 47-LDA-mouse IgG2a	Yes	Yes	n.d.	[17]
Cancer B cell lymphoma	NKG2D-Fc	Protein	Safe	Yes	Yes	Complement	[18]
Arthritis	Class II MHC-mIgG3	Protein	Induction of Ag-specific hyporesponsiveness	n.d.	Yes	Inhibits autoreactive T cells by cross-linking TCR	[19]
Encephalomyelitis	Myelin basic protein-mIgG1	Protein	Yes, increased T cell proliferative responses	n.d.	n.d.	FcRn dependent	[20]
Allergy	Fcγ-Fcε<br>Feld1-hIgG1-Fc<br>Fc-DARPin-hIgG1	Protein	Inhibitory	Yes	Inhibits mast cell and basophil function	Fc-dependent	[21-23]

n.d., not determined; mIgG, mouse IgG; hIgG, human IgG; s.c., sub-cutaneous; i.n., intranasal; i.p, intraperitoneal; i.v., intravenous; FCA, Freund's complete adjuvant; PfMSP1-19, *Plasmodium falciparum* merozoite surface protein 1-19; HSV, Herpes Simplex Virus; BoNT, Botulinum neurotoxin; CTLA4, cytotoxic T lymphocyte antigen; NKG2D, Natural Killer cell receptor G2D.

1. White, D.M. et al. Rapid immune responses to a botulinum neurotoxin Hc subunit vaccine through in vivo targeting to antigen-presenting cells. *Infection and immunity* **79**, 3388-3396 (2011).
2. Zaharatos, G.J. et al. HIV-1 and influenza antigens synthetically linked to IgG2a Fc elicit superior humoral responses compared to unmodified antigens in mice. *Vaccine* **30**, 42-50 (2011).
3. Lu, L. et al. A neonatal Fc receptor-targeted mucosal vaccine strategy effectively induces HIV-1 antigen-specific immunity to genital infection. *Journal of virology* **85**, 10542-10553 (2011).
4. Zhang, M.Y., Wang, Y., Mankowski, M.K., Ptak, R.G. & Dimitrov, D.S. Cross-reactive HIV-1-neutralizing activity of serum IgG from a rabbit immunized with gp41 fused to IgG1 Fc: possible role of the prolonged half-life of the immunogen. *Vaccine* **27**, 857-863 (2009).
5. Chen, H., Xu, X. & Jones, I.M. Immunogenicity of the outer domain of a HIV-1 clade C gp120. *Retrovirology* **4**, 33 (2007).
6. Obregon, P. et al. HIV-1 p24-immunoglobulin fusion molecule: a new strategy for plant-based protein production. *Plant biotechnology journal* **4**, 195-207 (2006).
7. Loureiro, S. et al. Adjuvant-free immunization with hemagglutinin-Fc fusion proteins as an approach to influenza vaccines. *Journal of virology* **85**, 3010-3014 (2011).
8. Konduru, K. et al. Ebola virus glycoprotein Fc fusion protein confers protection against lethal challenge in vaccinated mice. *Vaccine* **29**, 2968-2977 (2011).
9. Hong, Y. et al. Lentivector expressing HBsAg and immunoglobulin Fc fusion antigen induces potent immune responses and results in seroconversion in HBsAg transgenic mice. *Vaccine* **29**, 3909-3916 (2011).
10. Hong, Y. et al. Immunoglobulin fc fragment tagging allows strong activation of endogenous CD4 T cells to reshape the tumor milieu and enhance the antitumor effect of lentivector immunization. *J Immunol* **188**, 4819-4827 (2012).
11. Ye, L., Zeng, R., Bai, Y., Roopenian, D.C. & Zhu, X. Efficient mucosal vaccination mediated by the neonatal Fc receptor. *Nature biotechnology* **29**, 158-163 (2011).

12. Du, L. et al. A recombinant vaccine of H5N1 HA1 fused with foldon and human IgG Fc induced complete cross-clade protection against divergent H5N1 viruses. *PloS one* **6**, e16555 (2011).
13. Takashima, Y. et al. Immunization with pseudorabies virus harboring Fc domain of IgG makes a contribution to protection of mice from lethal challenge. *Vaccine* **23**, 3775-3782 (2005).
14. Mekhaiel, D.N. et al. Polymeric human Fc-fusion proteins with modified effector functions. *Scientific reports* **1**, 124 (2011).
15. Logan, G.J. et al. Antigen-specific humoral tolerance or immune augmentation induced by intramuscular delivery of adeno-associated viruses encoding CTLA4-Ig-antigen fusion molecules. *Gene therapy* **16**, 200-210 (2009).
16. Ferrone, C.R. et al. Adjuvanticity of plasmid DNA encoding cytokines fused to immunoglobulin Fc domains. *Clinical cancer research : an official journal of the American Association for Cancer Research* **12**, 5511-5519 (2006).
17. Gil, M. et al. Targeting a mimotope vaccine to activating Fcgamma receptors empowers dendritic cells to prime specific CD8+ T cell responses in tumor-bearing mice. *J Immunol* **183**, 6808-6818 (2009).
18. Zhang, B. et al. Immune Surveillance and Therapy of Lymphomas Driven by Epstein-Barr Virus Protein LMP1 in a Mouse Model. *Cell* **148**, 739-751 (2012).
19. Zuo, L. et al. A single-chain class II MHC-IgG3 fusion protein inhibits autoimmune arthritis by induction of antigen-specific hyporesponsiveness. *J Immunol* **168**, 2554-2559 (2002).
20. Mi, W. et al. Targeting the neonatal fc receptor for antigen delivery using engineered fc fragments. *J Immunol* **181**, 7550-7561 (2008).
21. Zhu, D. et al. A chimeric human-cat fusion protein blocks cat-induced allergy. *Nature medicine* **11**, 446-449 (2005).
22. Zhu, D., Kepley, C.L., Zhang, M., Zhang, K. & Saxon, A. A novel human immunoglobulin Fc gamma Fc epsilon bifunctional fusion protein inhibits Fc epsilon RI-mediated degranulation. *Nature medicine* **8**, 518-521 (2002).
23. Eggel, A. et al. Inhibition of ongoing allergic reactions using a novel anti-IgE DARPin-Fc fusion protein. *Allergy* **66**, 961-968 (2011).

Table S2. Summary of modifications

**Change heavy chain**	IgG2, IgG4	Lower affinity for FcγRs and complement receptors advantageous to therapeutic applications requiring low effector activation.
	IgG3	Activates complement and FcγR-mediated functions more efficiently than other IgG subclasses, although also more susceptible to proteolysis and exhibits a shorter half-life. However, a recent study identified an allotype that exhibits a considerably longer half-life (Stapleton et al, 2011).
	IgA, IgE, IgM	Possibly useful for vaccination strategies. IgA-ICs have been shown to lead to protection from mucosal pathogens (Bakema & van Egmond, 2011b). Studies with IgE mAbs suggest utility in cell-based tumor vaccines (Karagiannis et al, 2011). Naturally polymeric IgM may mimic the "depot-effect" of adjuvants (Czajkowsky et al, 2010), is involved in Ab subclass switching (Kavery et al, 2012), and has been shown to be an excellent adjuvant in vaccines (Harte et al, 1983).
**Modify lower hinge-Cγ2 domain**	Mutate	Location of binding sites of FcγRIIIA (and probably other FcRs) and C1q. Mutations that enhanced affinity to FcγRIIIA improved ADCC activity (Shinkawa et al, 2003; Stavenhagen et al, 2008).
	Lengthen	IgG3 has an extended hinge region. Other molecular extensions, such as SEEDbodies (Muda et al, 2011), are also possible.
**Mutate CH2/CH3 junction**	FcRn (Trim21)	Mutations that improved FcRn interaction also increased *in vivo* half-life and therapeutic efficacy (Zalevsky et al, 2010). Trim21 also binds within the same region and so mutations in this region will also likely influence antibody-dependent intracellular neutralization by Trim21 (McEwan et al, 2011).
**Modulate glycosylation**	FcR	De-fucosylation increases affinity for FcγRIIIA (Shinkawa et al, 2003; Stavenhagen et al, 2008).
	IVIG	Efficacy results from interaction with inhibitory sialic acid receptors, including DC-SIGN and Siglec-2 (Anthony et al, 2011; Seité et al, 2010). Enhanced sialic acid content lowers dosage requirement (Mekhaiel et al, 2011a).
**Optimize fusion partner**	Therapy	Differences in the affinity or stoichiometry of the fused partner for transmembrane TNF-α believed responsible for differences in clinical response between etanercept and infliximab (Van den Brande et al, 2003).
	Vaccine	With monomeric fusions, use of an Ag that recognizes receptors on APCs would enable cross-linking with FcγRs, which is required for a well-balanced immune response. The fusion partner can also affect binding to FcγRs (Mekhaiel et al, 2011b).
**Increase valency**	Expected to be generally useful strategy to increase potency of therapy and enable binding to receptors that only bind polymeric ICs, for example FcRL4 & FcRL5. Successful strategies include biosynthetic hexameric complexes (Mekhaiel et al, 2011b) and IgM-based complexes (Ammann et al, 2012).	
